# P-608. Impact of Guideline-Recommended Antibiotic Selection on Acute Exacerbation of COPD (AECOPD) Outcomes at a Large Community Health System

**DOI:** 10.1093/ofid/ofaf695.821

**Published:** 2026-01-11

**Authors:** Brey Fure, Emily Herstine, Delaney Hart, Krista Gens

**Affiliations:** Allina Health System, Woodbury, MN; Abbott Northwestern Hospital, Minneapolis, Minnesota; Abbott Northwestern Hospital, Minneapolis, Minnesota; Abbott Northwestern Hospital, Minneapolis, Minnesota

## Abstract

**Background:**

The Global Initiative for Chronic Obstructive Lung Disease (GOLD) guidelines recommend antibiotics for AECOPD only in patients meeting criteria. Inappropriate antibiotic selection in patients with AECOPD can lead to increased readmission rates, increased length of hospital stays, and treatment failure.

**Figure:**
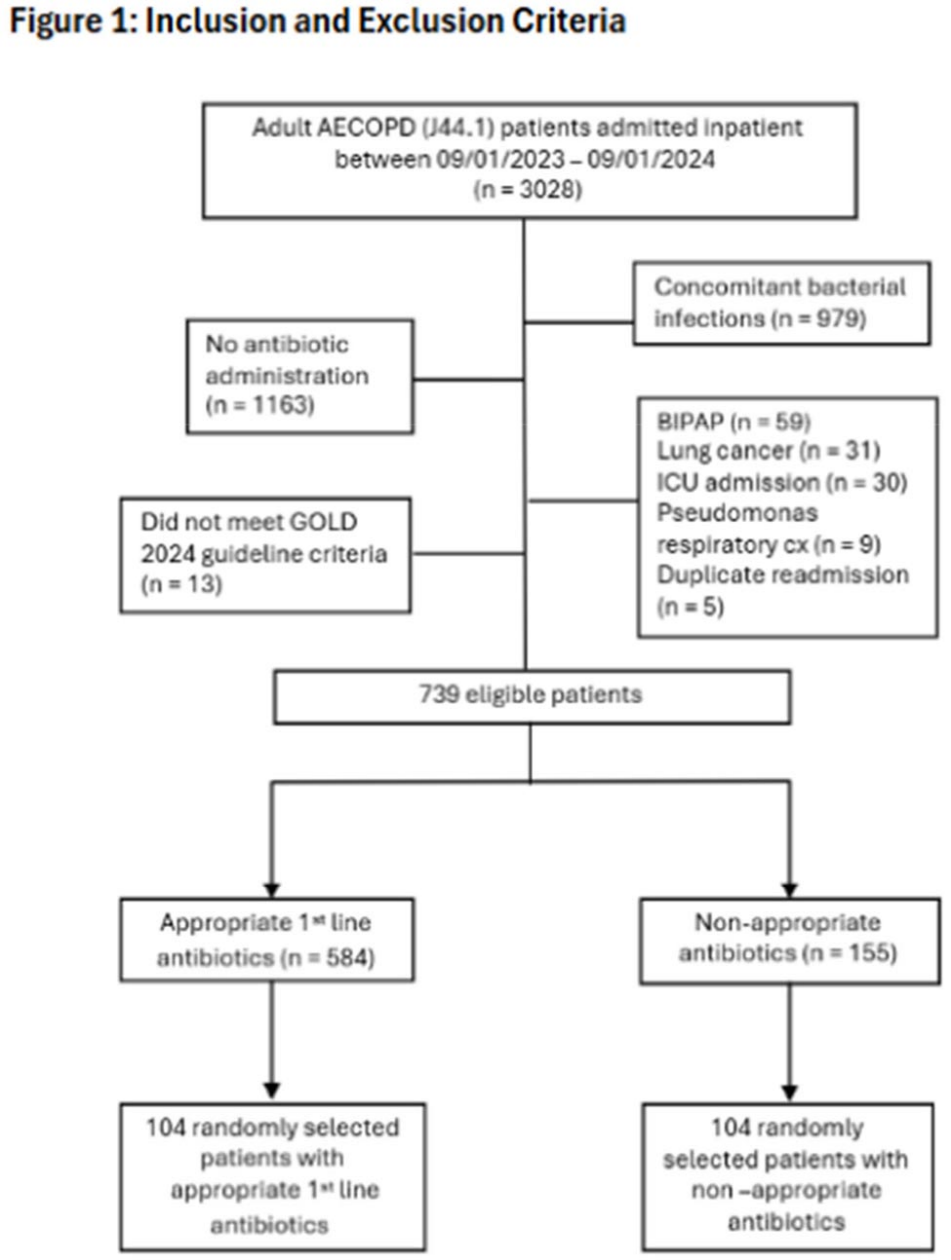

**Figure:**
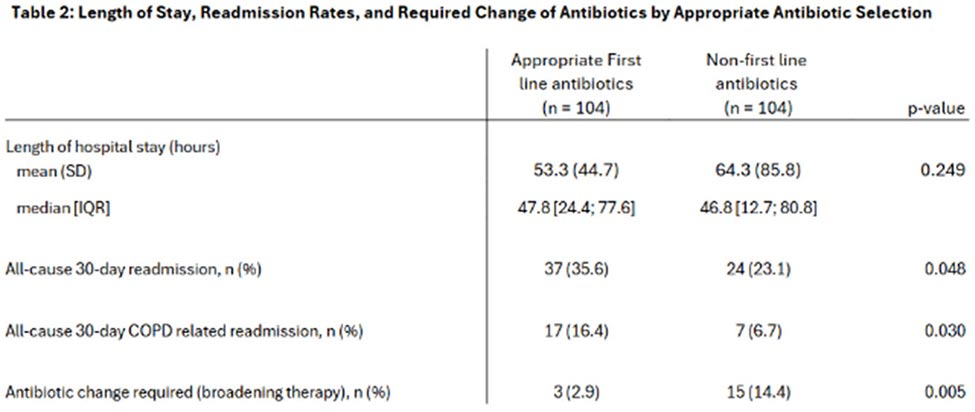

**Methods:**

A retrospective observational study was conducted of adult inpatients at a community health system between September 1, 2023, and September 1, 2024 with a primary diagnosis of AECOPD. Patients were excluded from this study as outlined in Figure 1. Appropriate 1st line antibiotic therapy was defined by the institution’s guideline. Patients initially receiving antibiotics other than appropriate 1st line therapy who transition to 1st line therapy within 24 hours were considered as having received appropriate therapy. The primary endpoint of this study was length of hospital stay (LOS), and secondary endpoints included all-cause 30-day readmissions, COPD-related readmissions, and the percentage of patients requiring a change in antibiotics.

**Results:**

In this study doxycycline was the primary appropriate 1st line antibiotic utilized (88.5%), while azithromycin was the predominately used non appropriate 1st line antibiotic (89.4%). Patients receiving appropriate 1st line antibiotics had a median LOS of 47.8 hours, compared to 46.8 hours for non-appropriate 1st line antibiotics (p=0.249). Patients with non-appropriate 1st line antibiotics had lower rates of all-cause 30-day readmissions and COPD-related readmissions. Patients treated with appropriate 1st line antibiotic therapy had a significantly lower rate of therapy requiring broadening (2.9% vs. 14.4%, p=0.005). Limitations include inability to fully assess adherence to GOLD guideline criteria (e.g. purulent sputum) and COPD stage.

**Conclusion:**

Guideline recommended antibiotics had mixed results with less patients requiring changes in therapy, but no difference in LOS, and higher rates of readmissions, which may have been impacted by study limitations.

**Disclosures:**

All Authors: No reported disclosures

